# Focal Infection
*A paper read before the Bristol Medico-Chirurgical Society on Wednesday, 14th March, 1934.


**Published:** 1934

**Authors:** A. J. M. Wright

**Affiliations:** Surgeon to the Nose and Throat Department, Bristol General Hospital


### FOCAL INFECTION.*

BY

A. J. M. Wright, F.R.C.S.,

*Surgeon to the Nose and Throat Department, Bristol General Hospital.*

---

It would seem advisable, as a first step, to define what I mean by focal infection. However desirable this may be, I find it difficult to do so, as the more one considers the subject the less capable of definition does it appear to be. As a basis, however, for these remarks I suggest that by focal infection I mean chronic disease in organs or tissues of the body directly resulting from a centre or centres of infection situated at a distance. This definition, no doubt, lacks scientific precision, but so does our knowledge. That much chronic disease is due to focal infection I firmly believe, but my purpose to-night is not to attempt to convert the unbeliever. Instead, I propose to place before you, by briefly relating illustrative cases, what I believe to be important points in the application of this principle to practice.

My first illustration represents the type of case in which an obvious infective focus exists, but owing to its not having been looked for irreparable damage has been done.

* A paper read before the Bristol Medico-Chirurgical Society on Wednesday, 14th March, 1934.

The patient, a woman aged 69, gave a history of chronic conjunctivitis and dacrocystitis of many years' standing. In addition she had an anterior polar cataract in one eye and commencing changes in the vitreous of her other, and only useful, eye. When questioned, she also gave a history of chronic sore throats for at least fifteen years, with left frontal headaches for about the same period. Examination disclosed a chronic suppuration in the left antrum, secondary to an old buried tooth fragment, and drainage of the antrum with removal of the tooth has resulted in some improvement in the vision of her seeing eye.

In contra-distinction to the case just quoted, in which the focus, when looked for, is very obvious, I should wish to illustrate a not uncommon type in which the opposite obtains, and in which the focus, far from being obvious, requires perhaps to some extent the eye of faith to recognize it. As an example of such a case, in which serious secondary results have followed a barely discoverable infective focus, I cannot do better than quote the following:—

A woman, aged 32, had suffered for some months from a pustular eruption on the face. When first seen, nearly eighteen months ago, she gave a history of progressive œdema of a few weeks' standing, the urine being solid with albumen. Careful investigation of the medical history disclosed nothing beyond a vague history of occasional sore throats, in no case sufficiently severe to lay the patient up. Superficial examination of the pharynx revealed nothing abnormal, but retraction of the anterior pillars disclosed small and somewhat unpleasant-looking tonsils. Removal of these has resulted in complete resolution of both the nephritis and the dermatitis.

Seeing that the focus of infection is frequently so difficult to identify, it is not surprising that one sometimes makes a mistake as to its situation. As an example of such a mistake of my own I will quote the following:—

The patient, a man aged 27, had suffered from recurrent attacks of iritis for three years, accompanied by a general neurasthenic condition.

Eighteen months ago I operated on his nasal septum, on the mistaken assumption that the iritis was secondary to a nasal infection. Six months later he developed a quinsy which was immediately followed by an attack of iritis, and after this there was a succession of sore throats followed on each occasion by a flare-up in the eyes. His tonsils were removed four months ago, and as to the result I cannot do better than to quote from a letter I recently received from him: "I can honestly say that ever since the operation I have never looked back, but have got slowly and steadily better every day and in every way. The eye attacks have each time become less, the wretched 'back of the neck' pain has been gradually becoming less until the last three weeks it has entirely gone, my energy is trebled, I have gained 12 lbs. in weight, the things I used to worry about no longer worry me—in other words, I have changed from a nervous wreck into a person that is full of energy and ready to battle with the next emergency."

In this case I have no doubt that the tonsillar infection was the primary one. As a result of experience I am now of the opinion that where one is in doubt as to the relative blame of possible foci in the nose and throat, the tonsils are by far the most frequently blameworthy.

Another somewhat similar case is the following:—

A male, aged 52, when first seen nine years ago was suffering from a chronic infection of the skin and subcutaneous tissue of the face, which he stated he had had for twenty years. His antra were dark on transillumination, and I then regarded the skin condition as being probably secondary to a chronic antral suppuration. Exploratory puncture, however, proved negative, and I did not see him again until six months ago. I then found him to be in much the same condition, but in light of further experience I paid more attention to his throat, and found that he had enlarged and obviously grossly infected tonsils. Removal of these has resulted in a rapid clearing up of the skin infection. In my experience chronic skin infections are much more frequently met with in association with infection in the tonsils than in the nose.

A not uncommon happening is to observe a case in which an infective focus is discovered and dealt with surgically with improvement in the secondary lesions, to be followed, however, at a later date by evidence of the arising of another focus of infection followed by a recrudescence of the secondary lesions.

As an example of this occurrence of consecutive foci, a female, aged 44, was seen five years ago with a history of nasal discharge of some years' standing, associated recently with severe muscular rheumatism. Drainage of infected nasal sinuses caused a complete clearing up of the rheumatism, with considerable improvement in the general condition. Four years later, however, she had an attack of acute tonsillitis, which was followed by a recurrence of the rheumatism. Removal of her tonsils has resulted in improvement in her general condition and rheumatic symptoms, but the operation has been performed too recently to judge of the final result.

Not uncommonly, however, instead of one focus following another, we find that multiple infective foci are present from the beginning, and the discovery of one may prevent a search being made for another which may be of even greater importance.

This is exemplified by a woman, aged 42, who fifteen years ago was affected with septic arthritis of relatively sudden onset. This was presumed to be associated with infected teeth and these were dealt with, with perhaps some degree of improvement, but the patient remained more or less an invalid. The occurrence of an otitis media a year ago led, as a routine, to the examination of the nose. To my surprise I found on transillumination that the antrum on one side was completely opaque. Operation disclosed chronic disease in the antrum, which from its nature had almost certainly resulted from a dental infection many years previously, and a recent note of her condition states that she is now fit, fat and well. Some deformity of the joints remains, but no sign of any active disease.

One of the most difficult classes of case with which we have to deal is that in which allergy, hypersensitiveness, or whatever term one uses, appears to be a pronounced factor. This, at present, somewhat nebulous portion of the machinery of the reaction of the body to infections seems to be obtaining an increasing share of attention.

As an example of such a case, I quote a male, aged 47, whom I first saw eleven years ago for a slight nerve deafness, tinnitus, and attacks of sneezing and watery nasal discharge. This condition persisted in spite of vaccines and other treatment for seven years, when removal of infected teeth was followed by an entire disappearance of the nasal symptoms. For a year before the teeth were dealt with the patient had, in addition, an arthritis of the wrists, feet and ankles. Removal of the teeth was followed comparatively rapidly by the disappearance of the nasal symptoms, and the arthritis also cleared up, but not for an interval of about a year. This patient, who is a schoolmaster, and in spite of this is of an unusual grade of intelligence, tells me that he now has simply some slight residual stiffness of the joints, that he gets hay fever every summer, and that, curiously, the ingestion of either spinach or tomatoes is invariably followed by some recurrence of pain in the joints. Here we have, it seems to me, an individual who is constitutionally hypersensitive to many substances, including the products of focal infection around his teeth. In such a case the hypersensitiveness is far from being a contra-indication to the removal of an infective focus, but is rather the reverse. The case also illustrates what I believe to be a very important fact in deciding the prognosis in cases of focal infection. This fact is that one must let at least a year elapse before one can judge as to how far removal of the focus may have altered what are believed to have been secondary lesions.

A considerable amount of evidence is accumulating to suggest that derangements of the endocrine system are not infrequently due to focal infection. Among such, affections of the thyroid gland may be associated with a focus of infection which, in my somewhat one-sided experience, is usually tonsillar. In the discussion at the last meeting, the question was raised as to whether in a case of focal infection with a secondary thyroid derangement, the former or the latter should receive surgical attention. I would certainly agree that in cases presenting much evidence of toxicity the thyroid claims priority. On the other hand, although my experience is very limited, I believe that if attention to the infective focus is given at an early stage, the thyroid lesion will be checked.

As an example of this, a woman, aged 23, following a double quinsy preceded by frequent sore throats, developed some enlargement of the thyroid with some slight degree of toxicity and loss of weight. Removal of her tonsils has resulted in a decrease in the thyroid enlargement and an increase in the woman of one and a half stone!

As another example of the effect of focal infection on the endocrine system I should instance the case of a woman, aged 34, who was seen some two years ago with enlarged and obviously infected tonsils and a history of recurrent iritis. She had also put on a considerable amount of weight, and was lethargic and irritable with loss of sexual impulses. Following tonsillectomy she has become a changed individual. The trouble in the eyes has cleared up, she has lost two stone in weight, she has become active and cheerful, and her sexual life is now normal.

Finally, two examples of the changes which may be produced in the nervous system by what is apparently a relatively innocent infection.

A male, aged about 47, and in a very responsible and rather worrying position, had felt tired and depressed with headaches for the best part of a year. In spite of prolonged rest, no improvement took place, and so serious was his condition that it seemed probable that he would have to give up his work. Thorough investigation disclosed no organic lesion beyond a doubtful degree of dental infection associated with crowned teeth, etc. Radical dental treatment resulted in complete recovery, and the individual is now fitter in every way than he has been for many years.

As an example of the effect of focal infection on a peripheral nerve, in contra-distinction to the central nervous system, I should like to illustrate a case in which the auditory nerve was the one to suffer. We all appreciate that the auditory nerve is relatively easily damaged. We expect it to degenerate early as a result of age changes, and we find that it is easily affected by chemical substances circulating in the blood, *i.e.* such drugs as quinine or salicylates. Cases of unexplained nerve deafness are, I believe, not infrequently to be explained as resulting from the toxic effects of a focal infection. What was to me a dramatic illustration of this is presented by the following case :—

A male, aged 52, was first seen by myself twenty-one years ago, and has been seen at odd intervals ever since. His hearing during the last ten years had gradually depreciated owing to a nerve defect. Some eighteen months ago I suggested that he should have his mouth, which was obviously infected, put in order. I did not see him again until recently, when, to my surprise, I found that his hearing had recovered to a degree which brought it back to what it had been some ten years previously. Apart from the removal of his infected teeth, I discovered no other possible factor which could have produced this improvement.

To sum up. If one is a believer in focal infection, as you appreciate that I most surely am, it is of paramount importance in this, as in other branches of medicine, that examination should be thorough if mistakes are to be avoided. In the majority of cases there is, at present, no satisfactory alternative to dealing with the focus surgically. In regard to this it is perhaps of interest to quote from the report of the British Medical Association Committee on arthritis. They state that surgical treatment of an infective focus may fail because it was not the guilty one, because another focus may exist, such as an endocrine one, following a tonsillar or dental one, because a state of hypersensitiveness may persist, or, finally, I suggest, but they do not, because the removal of the focus has not been carried out sufficiently early in the history of the case, *i.e.* before the secondary results have become too firmly established to clear up.

